# Ecological Response of Pondweeds (*Potamogeton* and *Stuckenia*) to Water Physical and Chemical Parameters in Croatia (Southeastern Europe)

**DOI:** 10.3390/plants15060889

**Published:** 2026-03-13

**Authors:** Marija Bučar, Anja Rimac, Vedran Šegota, Nina Vuković, Antun Alegro

**Affiliations:** Division of Botany, Department of Biology, Faculty of Science, University of Zagreb, Marulićev trg 20/II, 10000 Zagreb, Croatia; marija.bucar@biol.pmf.hr (M.B.); vedran.segota@biol.pmf.hr (V.Š.); nina.vukovic@biol.pmf.hr (N.V.); antun.alegro@biol.pmf.hr (A.A.)

**Keywords:** aquatic plants, bioindication, ecology, macrophytes, Water Framework Directive, water parameters

## Abstract

Pondweeds, an important component of macrophyte vegetation, are influenced by various ecological factors of the aquatic ecosystem. In turn, pondweeds affect the nutrient and sediment dynamics and provide food and shelter for other organisms. As different species have specific environmental preferences and tolerances, they can serve as indicators of the ecological status of water bodies. Here, the ecological preference of the seven most frequent pondweeds in Croatia (*Potamogeton berchtoldii*, *P. crispus*, *P. lucens*, *P. natans*, *P. nodosus*, *P. perfoliatus* and *Stuckenia pectinata*) for chemical and physical water parameters was studied using 218 vegetation relevés and the accompanying water parameters. CCA revealed the main environmental gradients described by six parameters (chemical oxygen demand, total nitrogen, total phosphorus, electrical conductivity, dissolved oxygen and pH), while ecological responses of the species were further explored by GAMs. *Potamogeton berchtoldii*, *P. lucens*, *P. natans* and *P. perfoliatus* prefer clean, oxygenated, oligo- to mesotrophic water, and *P. crispus* and *S. pectinata* thrived in eutrophic water with low oxygen levels, while *P. nodosus* is a widespread generalist. The results of this study explain the distribution patterns of *Potamogeton* and *Stuckenia* species in Croatia, and add to the general knowledge on their role as bioindicators.

## 1. Introduction

The interaction between macrophytes and the aquatic ecosystem is complex and reciprocal. Macrophytes are influenced by hydrological and hydromorphological factors (flow velocity, water transparency, channel depth and width, substrate type and size), water physical and chemical parameters (electrical conductivity, pH, nutrient concentration, etc.) and biotic interactions such as competition for light and nutrients. On the other hand, macrophytes are key ecosystem engineers in an aquatic environment, modifying the physical and chemical conditions and hydrology and sediment dynamics, acting as primary producers and regulating nutrient dynamics, and providing habitat structures as shelter for other organisms in the ecosystem, shaping biodiversity patterns [1,2,3,4].

When discussing aquatic vegetation, lotic ecosystems must be addressed as heterogeneous and multidimensional, being influenced by processes acting in four interactive pathways—longitudinal (upstream-downstream movements), vertical (channel-underground aquifer interaction), lateral (influence from the riparian and floodplain zone) and time scale [5]. These shape and make the conditions in different reaches of the rivers quite diverse and changeable. Thus, rivers represent an inherently dynamic and unpredictable habitat, and this is made more pronounced and indeed exacerbated by increasing anthropogenic activity, especially that related to riverbed alteration such as channelisation, as well as the intensification of nutrient load and pollutant input. This kind of changing environment promotes highly competitive [6] and phenotypically plastic [7] species, as well as species tolerant to pollution [8].

The composition and abundance of aquatic plants reflect the biological quality of the aquatic environment, providing important insight into its ecological status, i.e., water quality and degree of hydromorphological alterations [9]. Low macrophyte diversity, the presence of invasive species, and species inclined to eutrophic habitats indicate the degradation of aquatic habitat from its reference condition. Certain macrophyte species are particularly sensitive to the changes in their environment, such as eutrophication or hydromorphological alterations, and are thus recognised as valuable and reliable ecological indicators. In many European countries, macrophytes are used as bioindicators for the ecological assessment of rivers under the Water Framework Directive (WFD)— the EU’s water resource management regulation [10]. Multiple monitoring systems were made to meet the requirements of the WFD [11,12,13,14,15,16,17,18,19], and all of them recognise several species of the genera *Potamogeton* and *Stuckenia* (pondweeds) as bioindicators for aquatic habitats.

The family *Potamogetonaceae* is a cosmopolitan family of submerged, rooted macrophytes whose species richness is highest in the Palearctic [20]. There are 22 *Potamogeton* and 4 *Stuckenia* species in Europe [21], as well as numerous hybrids [22,23,24,25,26,27]. Croatia is a part of Southern Europe, which includes the Mediterranean Basin, a diversity hotspot not only for aquatic plants [28] but for other organisms as well [29]. According to the Flora Croatia Database (FCD) [30], 15 *Potamogeton* species (including *Stuckenia pectinata* (L.) Börner, syn. *Potamogeton pectinatus* L.) and four hybrids have been recorded in Croatia. These species inhabit different types of water bodies in Croatia. In rivers, they avoid the turbulent, fast-flowing upper reaches where bryophytes dominate in aquatic vegetation [31]. In other river sections, different *Potamogeton* species can be part of the natural community, or they can indicate changes in trophic status [32] and changes in water-flow, i.e., the deceleration of water-flow, called ‘potamalisation’, as they tend to avoid rapid and shallow water, and prefer broader, slow-flowing lowland streams [22]. As an important part of the aquatic vegetation in rivers, pondweeds act as habitat engineers, influencing river hydromorphology by modifying flow velocity and sediment deposition, and increasing channel complexity and overall habitat heterogeneity [33].

This study encompasses the whole territory of Croatia and the inherent watercourse heterogeneity and investigates the ecological preferences of the most commonly occurring *Potamogeton* and *Stuckenia* species. We hypothesise that a gradient of physical and chemical parameters strongly influences the distribution of the selected pondweed species. The study aims to:

(1) identify the key physical and chemical water parameters that contribute most significantly to the distribution of selected *Potamogeton* and *Stuckenia* species,
(2) explore the ecological responses of selected *Potamogeton* and *Stuckenia* species and expand the existing data on their autecology and ecological preferences.

## 2. Results

### 2.1. Descriptive Statistics

**Figure 1:**
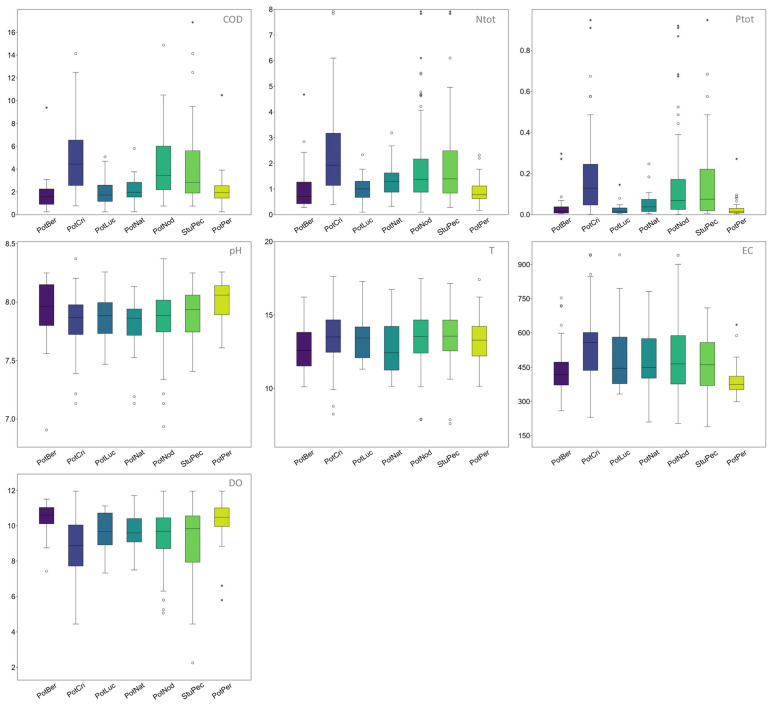
Descriptive statistics for studied *Potamogeton* and *Stuckenia* species. For variable abbreviation and measure units, see Table 1; species abbreviation: PotBer—*Potamogeton berchtoldii*, PotCri—*Potamogeton crispus*, PotLuc—*Potamogeton lucens*, PotNat—*Potamogeton natans*, PotNod—*Potamogeton nodosus*, PotPer—*Potamogeton perfoliatus*, StuPec—*Stuckenia pectinata*. Outliers—white circle > 1.5 SD; asterisk > 3 SD.

The seven most common *Potamogeton* and *Stuckenia* species with more than 15 records were included in the analysis: *Potamogeton berchtoldii* (35 records), *P. crispus* (78), *P. lucens* (18), *P. natans* (21), *P. nodosus* (140), *P. perfoliatus* (37) and *S. pectinata* (51). Descriptive statistics are presented in Figure 1 and in the Supplementary Materials, Table S1.

*Potamogeton berchtoldii* has the lowest medians for COD (1.58 mgO_2_/L) and Ntot (0.71 mgN/L), and among the lowest medians for Ptot (0.02 mgP/L) and T (12.59 °C). Also, *P. berchtoldii* has the highest median value for DO (10.6 mgO_2_/L), among the narrowest ranges for DO, and among the highest ranges for pH. The median value of *P. berchtoldii* for DO is significantly higher than that of all other species, except *P. perfoliatus* (Supplementary Materials, Table S2).

*Potamogeton crispus* has the highest median values for COD (4.43 mgO_2_/L), Ntot (1.93 mgN/L), Ptot (0.13 mgP/L) and EC (556.83 μS/cm), as well as the lowest median for DO (8.87 mgO_2_/L) and among the lowest medians for pH (7.87). The median values for Ntot and Ptot are significantly higher than those of all other analysed species, except *S. pectinata*, while the median for DO is significantly lower than for most species (Supplementary Materials, Table S2). In addition, *P. crispus* displays the broadest range for Ptot and among the broadest ranges for EC, DO, Ntot and T.

*Potamogeton lucens* has among the lowest median values for COD (1.73 mgO_2_/L) and Ptot (0.02 mgP/L). It also shows the narrowest ranges for COD, Ptot, T and DO.

*Potamogeton natans* has the lowest median values for pH (7.86) and T (12.43 °C). It also displays some of the narrowest ranges for COD, Ntot and Ptot.

*Potamogeton nodosus* is the most tolerant species as it has the broadest ranges for EC, Ntot, T and pH, and also among the broadest ranges for Ptot (after *P. crispus* and *S. pectinata*) and COD (after *S. pectinata*). Similar to *P. nodosus*, *S. pectinata* is ecologically very tolerant to the measured variables, exhibiting the broadest ranges for COD and DO, and some of the broadest ranges for Ntot, Ptot and T. It has the highest median value for T (13.55 °C). Additionally, there are no significant differences between the median values of *S. pectinata* and *P. nodosus* for any of the individual predictor variables (Supplementary Materials, Table S2).

*Potamogeton perfoliatus* has the highest median value for pH (8.06) and second-highest median value for DO (10.48 mgO_2_/L). *Potamogeton perfoliatus* has the lowest median values for Ptot (0.01 mgP/L) and EC (374.71 μS/cm), the second-lowest median values for Ntot (0.78 mgN/L) after *P. berchtoldii*, and the narrowest ranges for Ntot, EC and pH. The median values of *P. perfoliatus* for EC, DO and pH are significantly lower than those of all other species, with the exception of *P. berchtoldii* for DO and EC (Supplementary Materials, Table S2).

### 2.2. Canonical Correspondence Analysis and General Additive Models

**Figure 2:**
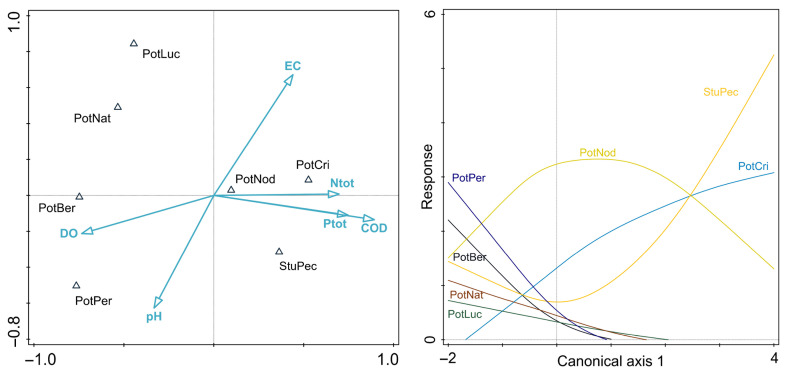
CCA biplot for species and environmental parameters (**left**) and species response curves (GAM) against the first canonical axis (**right**). For variable abbreviation and measure units see Table 1. Species abbreviation: PotBer—*Potamogeton berchtoldii*, PotCri—*Potamogeton crispus*, PotLuc—*Potamogeton lucens*, PotNat—*Potamogeton natans*, PotNod—*Potamogeton nodosus*, PotPer—*Potamogeton perfoliatus*, StuPec—*Stuckenia pectinata*.

Canonical correspondence analysis explained 11.57% of total variation (2.92). The explained fitted variation of the first axis was 57.74% and of the second axis 25.88% (Figure 2). The analysis was statistically significant (permutation test, 999 permutations, *p* = 0.001). The first axis shows a gradient from oxygenated, oligotrophic to mesotrophic watercourses on the left side to the watercourses with high nutrient and organic matter content. The negative part of both axes is related to the sampling sites with more alkaline water, i.e., that of higher pH. The second axis corresponds most with an increase in electrical conductivity. Species associated with the more oxygenated sites that have lower nutrient levels are *P. perfoliatus*, *P. berchtoldii*, *P. lucens* and *P. natans*. On the other hand, *P. crispus* is associated with eutrophic watercourses, as well as *S. pectinata*. *Potamogeton nodosus* occupies an intermediate position on the CCA ordination diagram, with a slight shift toward the eutrophic side of the gradient. *Potamogeton lucens* shows the strongest response along the second ordination axis, which primarily represents a gradient of electrical conductivity, with the species favouring higher conductivity values.

The species’ response to the observed compositional gradient was further elucidated with the general additive model of the first CCA axis. Here, the species positioned on the left side of the CCA ordination diagram consistently show a decline in relative abundance (RA) with increasing eutrophication and higher oxygen demand. On the other hand, *P. crispus* and *S. pectinata* increase in abundance with the increase in nutrient load, while *P. nodosus* is the only species with a unimodal response along the first axis.

**Figure 3:**
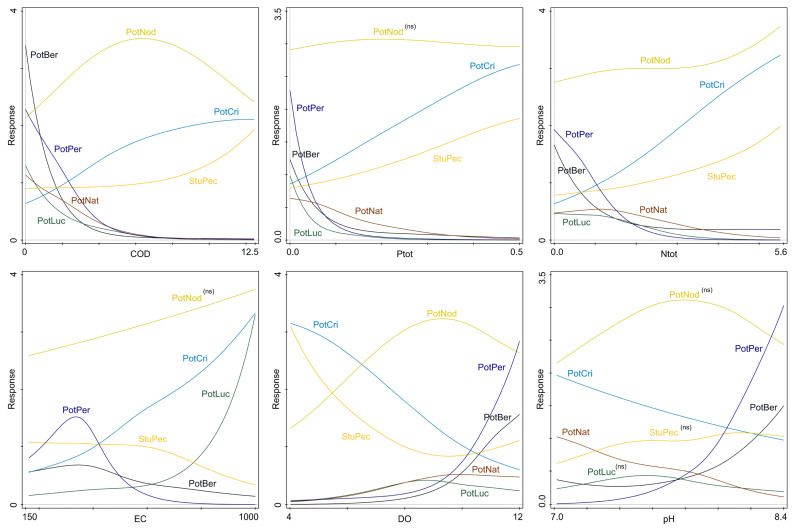
Generalised additive model (GAM) plots depicting species’ responses to individual predictor variables. The statistically non-significant models are marked with ‘(ns)’ next to the species abbreviation. For variable abbreviation and measure units see Table 1. Species abbreviation: PotBer—*Potamogeton berchtoldii*, PotCri—*Potamogeton crispus*, PotLuc—*Potamogeton lucens*, PotNat—*Potamogeton natans*, PotNod—*Potamogeton nodosus*, PotPer—*Potamogeton perfoliatus*, StuPec—*Stuckenia pectinata*.

The GAM response curves of the investigated *Potamogeton* species to the most influential environmental variables from the CCA additionally corroborated observed patterns (Figure 3). The GAMs for each variable are shown with axis ranges corresponding to variable values, excluding outliers. GAM could not generate the response of *P. natans* to EC. Additionally, the modelled responses of *P. nodosus* to Ptot, pH and EC, and the responses of *P. lucens* and *S. pectinata* to pH were statistically non-significant. The responses of all other species to individual predictor variables were statistically significant (*p* < 0.05) (Supplementary Materials, Table S3).

The distribution of *P. perfoliatus* is best explained by all predictor variables (R2 > 14%), compared to other pondweed species. It has a strong declining response to COD, Ntot and Ptot, an increasing response to pH and DO, and has a unimodal response curve for EC, with an optimum close to 350 μS/cm. *Potamogeton berchtoldii*, *P. natans* and *P. lucens* have declining responses to COD, Ntot and Ptot, comparable to those of *P. perfoliatus*. Moreover, the RA of *P. berchtoldii* follows the pattern of *P. perfoliatus* for all variables, except for COD, to which it is even more sensitive. However, the species has lower RA overall, compared to *P. perfoliatus*.

*Potamogeton natans* and *P. lucens* have similar responses regarding most variables. However, *P. natans* tolerates higher values of Ptot, up to 0.3 mgP/L, with a gradual declining response. By contrast, the RA of *P. lucens* exhibits a sudden drop from 0 to 0.1 mgP/L.

The RA of *P. crispus* shows a positive response to increasing values of Ntot, Ptot and EC, and a negative response to DO and pH, within the measured ranges of these variables. The RA of *P. crispus* plateaus at higher COD (10–12.5 mgO_2_/L) values.

Similar to *P. crispus*, *Stuckenia pectinata* also responds positively to increasing values of COD, Ntot and Ptot; however, its response to the gradient of EC is negative, especially in the higher end of the measured range (600–1000 μS/cm). Increasing values of DO affect the RA of *S. pectinata* negatively. The RA of *S. pectinata* shows a prominent decrease with the increase in DO, up to the value of around 9 mgO_2_/L, after which it exhibits a minimal, gradual increase.

As it is the most common species, *Potamogeton nodosus* has the highest overall RA in comparison to other investigated species. It has a unimodal response to most variables. Its wide unimodal curves, with optima close to the middle of the measured ranges, reveal the wide ecological tolerance of *P. nodosus*. An increase in Ntot values across the measured range has a slight positive influence on the RA of *P. nodosus*.

For clarity, the results are summarised in Table 1.

## 3. Discussion

Our findings indicate that water parameters play an important role in explaining the distribution of the investigated *Potamogeton* species in Croatia, with the most common species differentiated along gradients of Ptot, Ntot, COD, DO, as well as EC and pH. The importance of some of these parameters is supported by numerous studies. Budka et al. [34] found that among environmental variables, pH, BOD, PO_4_^3−^, NH_4_^+,^ and latitude influenced the vegetation structure of vascular macrophytes in lowland rivers of Poland with similar abiotic properties. Kennedy et al. [35] suggested that pH, alkalinity and nitrate concentration were among the main environmental drivers of macrophyte community composition and diversity in Zambia. El-Ghani et al. [36] investigated the most influential physical and chemical water parameters and demonstrated that pH, EC, NO_3_^−^, COD, BOD, Cu, Pb, Hg, SO_4_^2−^, HCO_3_^−^ and Ca affected the vegetation in the Nile Delta the most. Out of 10 measured water chemistry parameters in Turkey [37], water temperature, EC, DO, and pH best explained the distribution of *Potamogetonaceae* species. As BOD, orthophosphates and ammonium content are correlated to COD, Ptot and Ntot in this study, we can draw very similar conclusions.

The first axis of the CCA explains nearly half of the fitted explained variation, reflecting a gradient of nutrient enrichment and decline in dissolved oxygen, which can be indicative of eutrophication. Certain macrophyte species have already been associated with different trophic statuses. Thus, macrophytes can be used as ecological indicators and are one of the biological elements used to assess the water quality regarding eutrophication and river degradation [10]. Authors mostly agree that *P. crispus*, *S. pectinata* and *P. nodosus* are eutraphent species [22,34,38,39,40,41]. However, some discrepancies regarding the trophy preferences and ecological value of *Potamogeton* species are evident from the literature. *Potamogeton lucens* is associated with mesotrophic [41] to eutrophic water [39]. Although according to Schneider and Melzer [39], *P. natans* indicates mesotrophic conditions, Toivonen and Huttunen [38] have suggested it is indifferent to trophic status. This is supported by Zelnik et al. [41], who claim the association of *Potametum natantis* occurs in oligo- to eutrophic, but mostly in mesotrophic, waters. *Potamogeton perfoliatus* occurs from oligotrophic to eutrophic, but prefers eutrophic or meso-eutrophic conditions according to Preston [22], Toivonen and Huttunen [38] and Schneider and Melzer [39]. *Potamogeton berchtoldii* is known from meso-eutrophic water [39,42]. Although it is absent from oligotrophic habitats in Great Britain and Ireland [22], Gacia et al. [43] recorded *P. berchtoldii* in multiple oligotrophic lakes in the Pyrenees mountains. Our CCA result and the GAM of the first CCA axis resonate very well with the mentioned studies, excluding *P. perfoliatus*, which prefers water with low nutrient load.

The three most frequent pondweeds in Croatia, *P. nodosus*, *P. crispus* and *S. pectinata*, are all indicators of eutrophication and disturbance [34,40,41]. These species exhibited the highest medians and the widest ranges for Ntot, Ptot and COD in our study. However, *P. nodosus* stands out from the group with responses less extreme for COD, showing a unimodal pattern and optima at about 6.5 mgO_2_/L, with a decline in higher COD values where the relative abundances of *P. crispus* and *S. pectinata* still exhibit growth. A similar pattern was observed along the nutrient enrichment gradient represented by the first CCA axis. *Potamogeton nodosus* is among the most frequent macrophyte species in Europe [44,45,46], with a particularly high prevalence in Central and Southern regions. This widespread distribution underscores its ecological versatility and tolerance to diverse environmental conditions. This pattern may partly be explained by heterophylly, which is also observed in *P. natans*. The development of floating leaves provides these species with significant physiological and ecological advantages. Floating leaves enhance access to atmospheric CO_2_, increase direct interception of photosynthetically active radiation (PAR), and facilitate gas exchange at the water–air interface, while potentially shading competing submerged vegetation. At the same time, submerged leaves enable efficient nutrient uptake from the water column [47,48,49]. In a case study from the Tiber River, *P. nodosus* had an optimum in eutrophicated waters with high nutrient load in the middle and downstream sections of the river. In suburban areas, where EC, NH_4_^+^ and NO_3_^−^ values are high, and DO levels are low, *S. pectinata* dominated the aquatic vegetation [50]. *Stuckenia pectinata* and *P. crispus* clearly have great tolerance to nutrient enrichment and even thrive in eutrophic to hypertrophic, and often polluted water, as supported by numerous studies [34,40,41,50,51,52,53,54]. Our study shows that in Croatia, *P. crispus* occurs in the most extreme sites, exhibits the highest median values for COD, Ntot, Ptot and EC, and shows a positive response in RA with the increasing levels of these parameters. Consequently, it has the lowest median DO value. While *S. pectinata* exhibits a positive response to increasing COD, Ntot, Ptot values, it has a negative response to increasing EC. On the other hand, the RA of *P. crispus* increases in the measured ranges of this parameter within this study.

*Potamogeton lucens* occurs in more abundance in highly conductive watercourses in Croatia. It has a positive monotonic response to increasing values of EC. When the value of EC exceeds 650 μS/cm, only the RAs of *P. lucens* and *P. crispus* continue growing. This is in accordance with Preston et al. [22], who state that *Potamogeton lucens* prefers calcareous water in Great Britain and Ireland. Waterbodies on calcareous substrate can have hard and electrically conductive water due to the dissolved minerals from the substrate. In Pomerania (Poland), *P. lucens* was also positively influenced by high EC in watercourses, lakes and estuaries [55]. *Potamogeton lucens* is well adapted to hard, carbonate-rich, and eutrophic alkaline waters due to its capacity to efficiently utilise bicarbonate (HCO_3_^−^) as an alternative inorganic carbon source. Through the generation of pH gradients at the leaf surface, where acidification of one leaf side facilitates conversion of bicarbonate into CO_2_ for uptake, while the opposite side becomes alkaline, the species can maintain efficient photosynthesis under CO_2_-limited conditions [56,57,58,59]. This physiological mechanism provides a competitive advantage over the species restricted to or having a higher affinity to CO_2_, enabling the dominance of *P. lucens* in alkaline and carbonate-rich environments, as well as in dense stands where CO_2_ is especially scarce.

Nutrient load is known to negatively affect *P. lucens* [34,53], and Germ et al. [54] reported its presence only in the upper, less disturbed part of the Ljubljanica River in neighbouring Slovenia. In Croatia, it prefers sites with low nutrient levels, particularly total phosphorus, but it can still be found in sites with moderate levels. *Potamogeton lucens*, like *P. natans*, is described as a slow-growing, broad-leaved species affected negatively by the degradation and eutrophication of lowland rivers and streams during recent decades [60]. In contrast to our study, Jenačković et al. [61] found *P. lucens* unresponsive to EC and nutrients (NH_4_^+^ and PO_3_^4−^).

Apart from *P. lucens*, the species *P. perfoliatus*, *P. berchtoldii*, and *P. natans* prefer clean and well-oxygenated waters and are sensitive to eutrophication. Many sites where these species were recorded are located in the upper reaches, sometimes at higher altitudes, and are characterised by minimal disturbance and therefore cleaner water, with lower nutrient and organic matter content, high dissolved oxygen levels and a higher pH level. Most of these sites in Croatia are located in the Continental-Dinaric Ecoregion, which is characterised by karst landscape, built of calcareous and dolomite bedrock, hence the high water pH level. Since this area is also less inhabited due to the complex terrain filled with karst relief and the higher altitudes, the watercourses are less disturbed by human activity. Lacoul and Freedman [62] found that rivers at higher altitudes are generally less eutrophic than those in lowlands. Bobrov and Chemeris [63] stated that *P. berchtoldii* and *P. natans* are characteristic of the upper reaches of watercourses in the north of European Russia. There was a similar situation in the Ljubljanica River (Slovenia), where *P. natans* and *P. perfoliatus* were more abundant in the upper, less-disturbed part [54]. In central parts of western Ukraine, *P. natans* grows in the high-mountain areas of the Carpathians, and *P. berchtoldii* and *P. perfoliatus* grow just below, in the forest zone [64]. In Turkey, *P. berchtoldii* was most frequent at elevations above 800 m a.s.l., *P. natans* above 1200 m a.s.l., and *P. perfoliatus* was recorded across the whole altitudinal range, but most frequently in lowlands [37]. Multiple occurrences of *P. berchtoldii* and individual occurrences of *P. natans* and *P. perfoliatus* were documented in the mostly oligotrophic, high mountain lakes (above 2000 m a.s.l.) in the Pyrenees mountains [43]. Here, *P. berchtoldii* was positively associated with pH, as in our study, although the measured range in the lakes was 5.66–7.68, which is rather low compared to the 6.91–8.06 in our study. *Potamogeton berchtoldii* was also positively associated with conductivity, which was correlated with alkalinity; however, the lake water overall had very low conductivity (only up to 78.2 μS/cm). The interval of measured values for conductivity in our study does not overlap with the results of Gacia et al. [43], and is much broader (189.3–942.6 μS/cm), therefore elucidating its relationship to *P. berchtoldii* even further. Our study complements Gacia et al. [43] in that the RA of *P. berchtoldii* grows with increasing values of EC up to about 350 μS/cm where it reaches its peak, plateaus and decreases in values over 400 μS/cm. However, it should be noted that *P. berchtoldii* was formerly treated as a part of *P. pusillus* agg., and may have been misidentified in studies published before the clarification of the species within this group, potentially leading to misinterpretations of the species’ ecologies [65]. In the mentioned study [43], *P. natans* was present only in one eutrophic lake. The level of total phosphorus in this lake was 8.28 μM, which is equivalent to 0.256 mgP/L, approximately in the middle of the measured range of Ptot in our study.

Preston [22] reported that *P. natans* is, ecologically, the most tolerant species of the genus in Great Britain and Ireland, occurring across a wide range of habitats, from oligotrophic to eutrophic. However, *P. natans* showed very specific ecological requirements in Pomerania (Poland), inhabiting both watercourses and lakes under the same environmental conditions—well-oxygenated water with high redox potential [55]. Two studies highlight the species’ sensitivity to nutrient enrichment, which is in accordance with our study—*P. natans* showed a negative response to the increase in BOD in a study conducted in Polish watercourses [34], while in Northwestern Germany, the eutrophication trend from 1950s to 1990s resulted in a strong decline of *P. natans* frequency [53]. In the latter study, this was reported for *P. perfoliatus* as well. Interestingly, in that study, *P. berchtoldii* was positively affected by eutrophication, which is the opposite of our findings.

*Potamogeton perfoliatus* has the narrowest ecological range among the other pondweeds in Croatia. It is a species of non-eutrophic waters rich in dissolved oxygen and high pH. In support of this, Budka et al. [34] found that *P. perfoliatus* had low medians for NH_4_^+^, PO_3_^4−^ and BOD, but the highest median for pH value. Interestingly, this is in contrast to Preston [22], who says that in Great Britain and Ireland, *P. perfoliatus* has a wide ecological tolerance ranging from almost oligotrophic to eutrophic lakes and rivers. It is possible that *P. perfoliatus* found its niche in the less eutrophicated rivers of Croatia, growing more abundantly in the oligotrophic habitats where the competition is low, and avoiding the competition with congeners in the nutrient-rich watercourses.

Preston [22] reported that *P. berchtoldii* tolerates a wide range of pH values and nutrient conditions. This is in accordance with our study, as *P*. *berchtoldii* has the second broadest range for pH. However, although *P*. *berchtoldii* occurs in some eutrophic localities, which displays its tolerance to nutrient load, it seems to prefer mesotrophic and highly oxygenated environments in Croatia.

Even though species of the pondweed family separated well in the ordination analysis along the gradients of the selected water chemistry parameters, these variables explained a relatively low proportion (11.57%) of total variation. Similarly, Bayındır and İkinci [37] were able to explain only 5.9% of total variability using water chemistry. These results suggest that the distribution of macrophytes is influenced by multiple factors and highlight the need to consider additional environmental variables, such as mineral content [36,55], flow velocity [66,67], substrate characteristics [67,68,69,70], land use [69,71], and shading [35,67,72], to more clearly explain the distribution of aquatic plants in rivers. Therefore, future research should identify additional factors contributing to pondweed distribution in Croatia.

## 4. Materials and Methods

### 4.1. Study Area

The study area encompasses the whole territory of Croatia, which, according to the national methodology for macrophyte survey under the WFD monitoring [73], is divided into two biogeographical regions—the Pannonian Ecoregion and the Dinaric Ecoregion. The Pannonian Ecoregion represents the continental lowland part of the country, situated between three large rivers—the Sava, Drava and Danube. It consists mostly of alluvial and diluvial plains characterised by silicate Quaternary deposits and solitary mountain massifs up to 1200 m a.s.l. The climate is temperate, with warm summers and no dry season in most of the territory (Cfb) and hot summers predominantly in the eastern part (Cfa) [74]. The Dinaric Ecoregion encompasses the Dinarides and the coastal region of Croatia, and is further divided into two subecoregions—the Continental–Dinaric Subecoregion and the Mediterranean–Dinaric Subecoregion. As the Dinarides are characterised by karst, the whole ecoregion is built of Mesozoic carbonate bedrock. The Continental–Dinaric Subecoregion is characterised by a temperate climate with no dry season and warm summers (Cfb) and constant river discharge levels, while the Mediterranean–Dinaric Subecoregion is characterised by a temperate Mediterranean climate, with dry and hot summers (Csa) [74] during which some intermittent rivers dry out.

### 4.2. Sampling and Data Collection

Alongside other macrophytes, *Potamogeton* and *Stuckenia* species were recorded within the national surface water monitoring scheme conducted from 2016 to 2024. Macrophyte vegetation is monitored to assess the ecological status of water bodies, as required by the WFD [10]. Sampling was performed in 677 sites located on different watercourse types recognised by the typology in the national methodology for macrophyte sampling [73]. A map showing the distribution of the sites surveyed and analysed was created using QGIS Desktop 3.4.9 software. Vegetation relevés were made following the extended Braun-Blanquet scale (r = one individual, + = up to 5 individuals, 1 = up to 50 individuals, 2m = over 50 individuals, coverage < 5%, 2a = coverage 5–15%, 2b = coverage 15–25%, 3 = 25–50%; 4 = coverage 50–75%; 5 = coverage over 75%) [75,76,77]. The nomenclature follows Euro + Med [21].

Water parameters (abbreviations are given in Table 2) were measured monthly by an accredited laboratory (Water Institute Josip Juraj Strossmayer, Zagreb, Croatia) and their annual averages were used in the analyses.

### 4.3. Statistical Analyses

**Figure 4:**
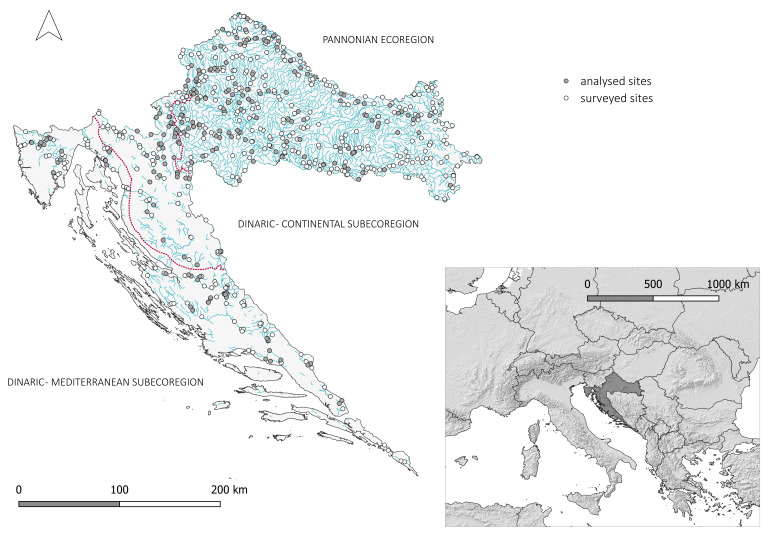
Study area with 677 surveyed sites and the 218 sites included in the analysis covering the whole Croatian territory (Southeastern Europe).

The *Potamogeton* and *Stuckenia* species recorded in more than 15 localities were further analysed. In total, 218 vegetation relevés, each from a different sampling site, were analysed (Figure 4). Most sampling sites are situated in the Pannonian Ecoregion (138), followed by the Mediterranean–Dinaric Subecoregion (43) and Continental–Dinaric Subecoregion (37). Regarding the hydromorphological alteration, 143 sites are natural, 56 significantly modified, and 19 artificial. The catchment areas of the watercourses at the investigated localities span from 2 km^2^ to more than 10,000 km^2^. These data on the sampling sites, alongside the species abundance, are listed in Supplementary Materials, Table S4. The distribution of the investigated *Potamogeton* and *Stuckenia* species along the gradient of water parameters was illustrated using descriptive statistics made in Past 5 software [78]. Furthermore, a significant difference between the water parameters for each species was tested with the Mann–Whitney pairwise post hoc test in Past 5 software [78].

To explore the relationship between water parameters and patterns in the distribution of the investigated pondweeds, a direct ordination method, canonical correspondence analysis (CCA), was used. This unimodal method was selected because the response data was compositional and had a 4.1 SD units-long gradient on the first axis. Physical and chemical parameters were tested beforehand for correlation using Past 5 software [78]. Due to high correlation statistics (Spearman’s rs ≥ 0.75, *p* < 0.05), only the following parameters were used for CCA: EC, pH, T, Ntot, Ptot, COD and DO. Due to the very low simple term effect and significance level of T (Supplementary Materials, Table S5), this predictor was left out of the final CCA biplot.

Generalised additive models (GAMs) were utilised to reveal the probability of occurrence of each species as a function of the water parameters used in the CCA. Poisson distribution was selected, with a logistic link function and two degrees of freedom. The Akaike Information Criterion (AIC) was used to select the model which best fits each response curve by modifying the smoothing parameter, which reduces the deviance [79,80]. When AIC detects that no candidate model has AIC values lower than the null model, it automatically removes the species from the model. The statistical significance of each model is shown in the Supplementary Materials, Table S3.

## Figures and Tables

**Table 1:** A summary of ecological preferences of the most common *Potamogeton* species in Croatia.

	Preferences for Water Physical and Chemical Parameters
*Potamogeton berchtoldii*	Thrives in oxygenated water with low nutrient content and high pH. Responds with a unimodal curve to EC (optimum at around 350 μS/cm). Generally similar ecological preferences to that of *P. perfoliatus*, but occurs in lesser abundance.
*Potamogeton crispus*	Hardy species which responds best to high nutrient load and EC. Very abundant even in poorly oxygenated water. Prefers less neutral water, pH close to 7.
*Potamogeton lucens*	Avoids heavy nutrient load, prefers mesotrophic water with moderate amounts of dissolved oxygen, and thrives in highly conductive watercourses.
*Potamogeton natans*	Prefers well-oxygenated, mesotrophic water with pH up to 7.8. Tolerates higher values of Ptot than other mesotrophic species (*P. berchtoldii*, *P. lucens* and *P. perfoliatus*).
*Potamogeton nodosus*	Most abundant and versatile species, tolerating both lower and upper parts of ranges, but thriving in the intermediate values of all measured parameters.
*Potamogeton perfoliatus*	Thrives in oxygenated water with low nutrient load and high pH. Responds with a unimodal curve to EC (optimum at around 350 μS/cm). Generally similar ecological preferences to those of *P. berchtoldii*, but occurs in higher abundance.
*Stuckenia pectinata*	Hardy species most abundant in low oxygen levels and thriving in highly eutrophic water. Unlike *P. crispus*, it prefers moderate levels of EC, and tolerates higher pH values.

**Table 2:** A list of analysed water parameters with their abbreviation and measurement unit.

Parameter	Abbreviation (Unit)
total alkalinity	ALK (mgCaCO_3_/L)
total hardness	TH (mgCaCO_3_/L)
total suspended solids	TSS (mg/L)
temperature	T (°C)
conductivity	EC (μS/cm)
total nitrogen	Ntot (mgN/L)
total phosphorus	Ptot (mgP/L)
nitrates	NO_3_^−^ (mgN/L)
nitrites	NO_2_^−^ (mgN/L)
ammonium	NH_4_^+^ (mgN/L)
ortophosphates	PO_4_^3−^ (mgP/L)
biochemical oxygen demand	BOD (mgO_2_/L)
chemical oxygen demand	COD (mgO_2_/L)
dissolved oxygen	DO (mgO_2_/L)
reaction	pH

## Data Availability

The original contributions presented in this study are included in the article/Supplementary Materials. Further inquiries can be directed to the corresponding author.

## Supplementary Materials

The following supporting information can be downloaded at: https://www.mdpi.com/article/10.3390/plants15060889/s1, Table S1: Descriptive statistics for analysed parameters per species; Table S2: Mann–Whitney U test of significant difference between species for measured water parameters; Table S3: Summary of fitted generalised additive models; Table S4: Hydromorphological data and species abundance across sampling sites; Table S5: Simple term effects of predictor variables.
